# Detailed analysis of retinal morphology in patients with diabetic macular edema (DME) randomized to ranibizumab or triamcinolone treatment

**DOI:** 10.1007/s00417-017-3828-1

**Published:** 2017-10-28

**Authors:** Sonja G. Karst, Jan Lammer, Christoph Mitsch, Manuela Schober, Janhvi Mehta, Christoph Scholda, Michael Kundi, Katharina Kriechbaum, Ursula Schmidt-Erfurth

**Affiliations:** 1 Department of Ophthalmology and Optometry, Medical University Vienna, Vienna, Austria; 2 Jaslok Hospital and Research Centre, Mumbai, India; 3 Center of Public Health, Medical University Vienna, Vienna, Austria

**Keywords:** Diabetic retinopathy, Diabetic macula edema, Ranibizumab, Triamcinolone, OCT, Fluorescein angiography

## Abstract

**Purpose:**

Our purpose was to compare the impact in diabetic macula edema (DME) of two intravitreal drugs (0.5 mg ranibizumab vs. 8 mg triamcinolone) on changes in retinal morphology in spectral-domain optical coherence tomography (SD OCT) images, color fundus photography (CF) and fluorescein angiography (FA) images during a 1-year follow-up.

**Methods:**

Post hoc analysis was conducted of morphologic characteristics in OCT, FA and CF images of eyes with a center involving DME that were included in a prospective double-masked randomized trial. Eligible patients were divided at random into two groups receiving either pro re nata treatment with 0.5 mg ranibizumab or 8 mg triamcinolone after a fixed loading dose. OCT and CF images were acquired at monthly visits and FA images every three months.

**Results:**

Twenty-five eyes of 25 patients (ranibizumab: *n* = 10; triamcinolone: *n* = 15) were included in this study. Patients treated with ranibizumab showed better visual acuity results after 12 months than patients receiving triamcinolone (*p* = 0.015) although edema reduction was similar (*p* = 0.426) in both groups. The initial effect on macular edema shedding after a single ranibizumab injection could be amplified with the following two injections of the loading dose. After a single injection of triamcinolone the beneficial initial effect on the macula edema faded within 3 months. Subretinal fluid and INL cystoid spaces diminished early in the course of treatment while fluid accumulation in the ONL seemed to be more persistent in both treatment arms. In FA, the area of leakage diminished significantly in both treatment arms. After repeated injections the morphologic OCT and FA characteristics of the treatment arms converged.

**Conclusions:**

Despite the higher dosage of triamcinolone, both therapies were safe and effective for treating diabetic macular edema. Fluid accumulation in the INL and subretinal space was more responsive to therapy than fluid accumulation in the ONL.

Clinicaltrials.gov: NCT00682539.

## Introduction

Diabetic macular edema (DME) is a leading cause of visual impairment in most developed countries [1]. Considering the increasing number of patients with diabetes, ocular complications are set to become a serious health issue in the near future [2]. Consequently, current treatment strategies for DME should be assessed in every possible detail.

DME has for a long time been graded exclusively in funduscopy as focal or clinically significant depending on its distance from the fovea. The advent of fluorescein angiography (FA) and optical coherence tomography (OCT) allowed DME to be characterized in detail [3, 4]. A great variety of morphology patterns of DME became apparent, even though all patients had the same underlying disease [5, 6]. Hence, a combination of OCT with FA is considered more advantageous for the classification of patients with diabetic retinopathy (DR) [7]. To date, little is known about the different pathophysiologic pathways that are directly associated with the distinct appearance in OCT or FA and their impact on disease progression and treatment response. Nevertheless, central retinal thickness (CRT) has been internationally established as the main morphologic marker in DME diagnosis and treatment follow-up regardless of the concurrent intraretinal changes.

Within the past decade, various multi-center studies have proven the effectiveness of intravitreally applied anti-vascular endothelial growth factor (VEGF) agents or corticosteroids in the treatment of DME [8–10]. In addition to a gain in visual acuity, CRT has been found to decrease to normal in most patients after repeated treatment. However, changes in morphologic characteristics have rarely been described as precise grading has not yet been automated and is therefore very time consuming.

This study investigated the detailed morphologic changes seen on multiple high-resolution cross-sectional OCT images of the macula, color fundus photography and standardized findings in early and late phase FA images of the macula in the course of pro re nata (PRN) treatment of DME with either ranibizumab, that had been approved by the FDA and the European Union for the treatment of vision impairment secondary to DME, or triamcinolone, that was used off-label.The dosage of triamcinolone was doubled to 8 mg/0.1ml in order to obtain a prolonged effect on DME resolution.

## Methods

Here, we report the results of the comparison of 0.5 mg ranibizumab and 8 mg triamcinolone PRN treatment for macula centers involving DME. The results of the first part of the study protocol comparing 2.5 mg bevacizumab and 8 mg triamcinolone have been published [11].

### Study design and participants

This prospective randomized double-masked clinical trial was conducted adherent to the 1964 Helsinki Declaration and its later amendments. It was registered at clinicaltrials.gov (NCT00682539) and approved by the responsible ethics committee of the Medical University Vienna (MUW; EK 363/2007) as well as by the Austrian Agency for Health and Food Safety (AGES). All procedures performed in this study were in accordance with the ethical standards of the institutional research committee.

Patients were recruited at the tertiary referral clinic for diabetic retinopathy at the Department of Ophthalmology, MUW. Written informed consent was obtained from each patient prior to study inclusion. Only one eye of each patient could be included in the study. Eligibility criteria were patients aged ≥18 years with type 1 or 2 diabetes, a best-corrected visual acuity (BCVA) between 20/25 and 20/400 (Snellen equivalent) and a macula center involving DME with a CRT of more than 300 μm measured with spectral domain (SD) OCT (Spectralis, Heidelberg Engineering GmbH, Dossenheim, Germany). Blood samples were drawn at baseline to evaluate glycemic control and preexisting comorbidities such as hypercholesterolemia or renal failure. Exclusion criteria were any vision-impairing ocular conditions other than diabetic retinopathy, a recent history of intraocular surgery or laser therapy within 3 months prior to study inclusion. If both eyes were eligible, the eye with the worse BCVA was included in the study.

### Intravitreal intervention

Eligible patients were divided at random into two groups to either receive three monthly injections of 0.5 mg ranibizumab (Lucentis**®**, Novartis Pharma AG, Vienna, Austria) or one intravitreal injection of 8 mg triamcinolone acetonide (Volon A**®**, Dermapharm GmbH, Vienna, Austria) dissolved in a volume of 0.1 ml. The treating physician prepared triamcinolone acetonide for intravitreal injection by replacing the solvent agent benzyl alcohol with physiologic saline solution immediately before intravitreal application. The baseline injection of triamcinolone was followed by two sham procedures at months 1 and 2 to maintain patients’ masking. Sham procedures were only mimicked by conjunctival contact without penetration of the ocular globe after the same preinjection care. The treating physician was unmasked.

Beginning at month 3, all patients were reassessed monthly by a masked physician to see if they needed PRN treatment of their assigned study medication based on the following predefined retreatment criteria: evidence of intraretinal or subretinal fluid on OCT with a 100-μm increase in CRT or a decrease in BCVA >5 letters compared with any previous study visit. Ranibizumab injections could be applied monthly whereas triamcinolone could be injected no more than every 3 months intermitted by sham injections.

### Data collection

Experts in BCVA testing and OCT imaging masked to the patients’ treatment assignment tested visual function and assessed retinal layer thickness at each monthly visit (30 ± 7 days). BCVA was measured on a logarithmic scale of the minimum angle of resolution (logMAR) using ETDRS charts at a distance of 2 m. Eyes were examined with a slit-lamp, indirect ophthalmoscope and intraocular pressure (IOP) was measured (Goldmann Applanation Tonometry, Haag Streit GmbH, Wedel, Germany). SD OCT images of the macula (20°×20° horizontal macula pattern centered at the fovea, high resolution, 49 sections, ART 9) were acquired. Furthermore, 30° and 60° color fundus photos were taken monthly centered on the fovea and on the optic disc (FF 450 plus camera, Carl Zeiss Meditec, Jena, Germany).

### Data assessment

OCT, fundus photo and FA image graders were masked to patients’ treatment assignment. CRT values were verified by reviewing the OCT scans for segmentation errors at the inner limiting membrane (ILM) and retinal pigment epithelium (RPE) and correcting them manually, if necessary using the HRA/Spectralis viewing module version 6.0.9. The ETDRS grid was centered manually for accurate CRT measurements when it was not centered on the fovea.

**Fig. 1:**
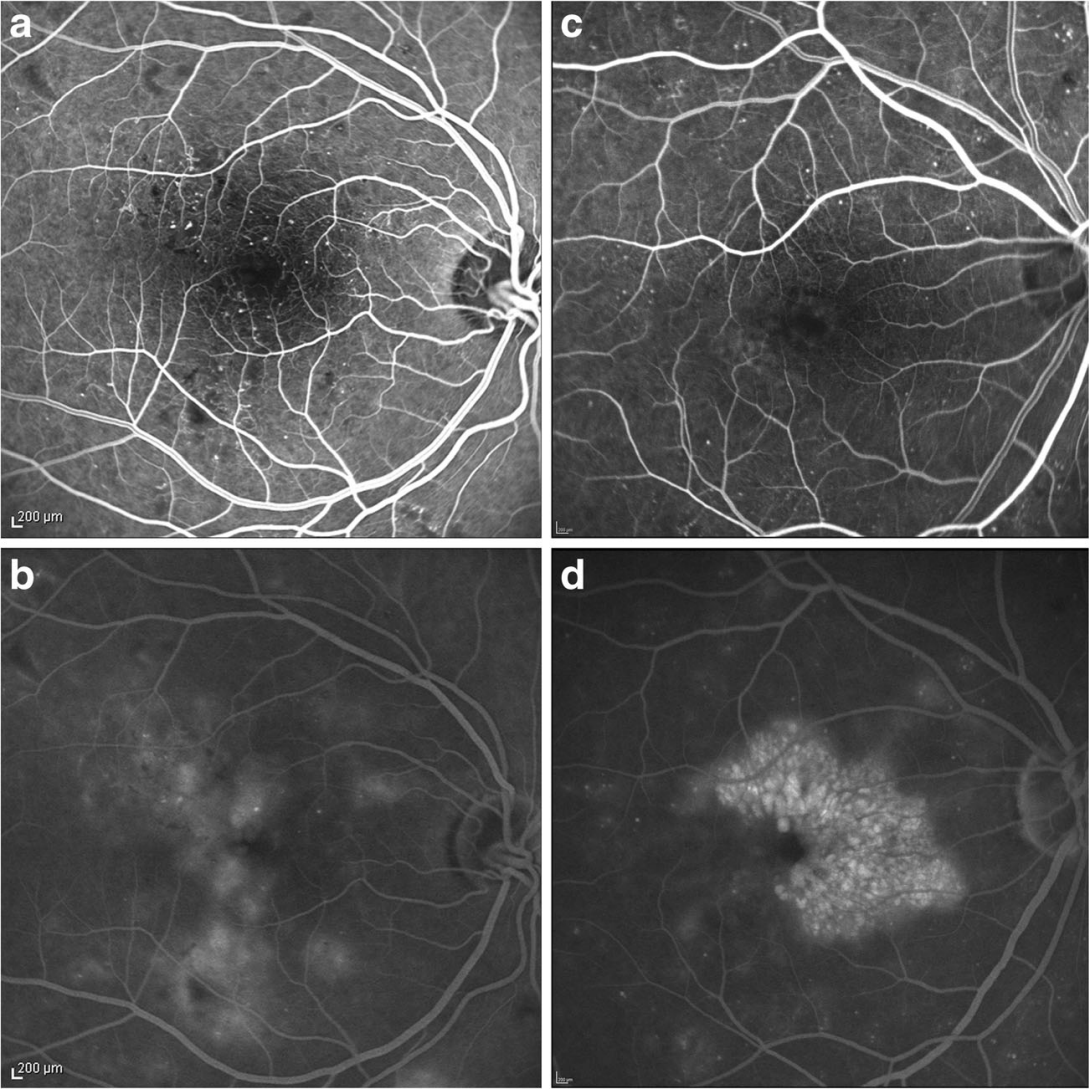
Fluorescein angiography leakage pattern: Images of the right eye of two patients diagnosed with diabetic macular edema. **a:** Early phase image showing several microaneurysms (MA) that are the source of leakage seen in the late phase image (**b**). Leakage predominantly (>50%) arises from microaneurysms and leakage pattern in **b** was classified as diffuse. **c:** The early phase image revealing hardly any MA. Leakage recorded in the late phase image **d** hardly arises from microaneurysms(<50%). The leakage pattern seen in **d** was classified as a honeycomb pattern

The morphology seen on OCT images and fundus photos acquired at baseline and 1,3,6 and 12 months after the first intravitreal injection were thoroughly graded. All 49 scans of the macular cube were graded for the presence of subretinal fluid (SRF), cystoid changes in the inner nuclear layer (INL) and cystoid- or sponge-like swelling in the outer nuclear layer (ONL), features that can be reliably detected in OCT scans [3, 12–14]. FA images at baseline and at month 12 were graded for signs of macula ischemia by manually outlining the area of the foveal avascular zone (FAZ) in early phase images using Spectralis software. Additionally, the area of fluorescein leakage was outlined manually and measured in late phase images accordingly. The source of leakage was assessed in early phase images in conjunction with late phase images using a modified grading based on the ETDRS report number 11: leakage predominantly (>50%) from microaneurysms or leakage hardly (<50%) from microaneurysms but from staining vessels [4] (Fig. 1). The pattern of hyperfluorescence secondary to dye pooling in cystoid spaces was classified as petaloid, honeycomb or diffuse in late phase images. [15] As DME can present different patterns in the same patient, all three patterns could be graded in parallel in the same FA image. Fundus photos were evaluated for diabetic retinopathy grading and the assessment of hard exudates (HE) and cotton wool spots (CWS).

### Statistics

The treating investigator allocated patients at random in permuted blocks stratified by sex, age and prior treatment at a 1:1 ratio to the study groups by using the online-randomization software provided by the Medical University Vienna (https://www.meduniwien.ac.at/randomizer/). The protocol specified that patients withdrawing their agreement to the study could be replaced up to one year before the projected study termination. The initial power analysis had to be repeated to account for the difference in sample size due to an imbalance in allocation that only became apparent after unblinding. The effect size that can be detected at the two-sided significance level of 5% with a power of 80% was a Cohen’s d of 1.05 for a 1:1 allocation ratio and 30 patients. This effect size increased to 1.23 with the 15:10 sample sizes. This effect size is still sufficient to detect a clinically relevant effect in changes of visual acuity during the year of observation.

The baseline characteristics of patients were compared across treatment groups by Fisher’s exact probability test for dichotomous, by the Fisher-Freeman-Halton test for categorical, and Mann-Whitney test for metric variables. Differences to baseline values of logMAR and retinal thickness were analyzed by general estimation equation (GEE) linear models with an ^^unstructured correlation matrix. Comparisons for each time point between treatment arms were done by linear contrasts applying Bonferroni-Holm correction. Within treatment groups, comparisons against baseline were done by testing the variable for the respective time point against zero. The correlation between BCVA and CRT was investigated by computing Spearman correlation coefficients. The relation between change in BCVA and change in retinal layers thickness was analyzed by linear regression. A corrected *p*-value of less than 0.05 was considered significant.

## Results

Of the 55 patients screened, 39 patients were included in the study. Twenty-five patients (ranibizumab *n* = 10; triamcinolone *n* = 15) continued the study follow-up throughout to the end of month 12, while 14 were lost to follow-up for compliance reasons. Epidemiologic baseline characteristics were similar in both treatment arms (Table 1). All patients (seven female) were diagnosed with type 2 diabetes with a mean duration of 14.2 ± 9.5 years and the mean age was 60.2 ± 15.1 years at presentation. About half of the patients were treatment naïve, while ten eyes had received prior laser and seven eyes prior intravitreal therapy for DME.

**Table 1:** Patients’ baseline characteristics

	Triamcinolone (*n* = 15)	Ranibizumab (*n* = 10)	*P*-value
Sex: female, n (%)	5 (33)	2 (20)	0.659
Age in years (±SD)	59.5 (18.6)	61.7 (8.5)	0.890
Pseudophakic n (%)	2 (13)	1 (10)	1.000
IOP in mmHg (±SD)	16.1 (3.7)	17 (2.5)	0.260
BCVA in logMAR (±SD)	0.34 (0.19)	0.34 (0.25)	0.375
CRT in μm (±SD) Spectralis OCT	481.9 (102.1)	516.2 (141.1)	0.267
Type 2 diabetes in n (%)	15 (100)	10 (100)	–
IDDM n (%)	8 (53.3)	6 (60)	1.000
Diabetes duration in years (±SD)	14.3 (10.3)	14 (8.7)	0.606
HbA1c (±SD)	7.5 (1.1)	7.5 (1.5)	1.000
Cholesterol (±SD)	179 (40)	180 (44)	0.997
Nephropathy: crea > 1.2% n (%)	2 (13)	4 (40)	0.175
Prior treatment n (%)			
Treatment naive	7 (47)	6 (60)	0.688
Focal/Grid laser	5 (33)	2 (20)	0.659
Panretinal laser	3 (20)	2 (20)	1.000
Anti-VEFG	4 (27)	2 (20)	1.000
Triamcinolone	1 (7)	1 (10)	1.000
DR severity n (%)			0.795
Mild NPDR	4 (27)	1 (10)	
Moderate NPDR	4 (27)	4 (40)	
Severe NPDR	5 (33)	4 (40)	
PDR	0	0	
QPDR	2 (13)	1 (10)	

*SD* standard deviation, *IOL* intraocular lens, *logMAR* logarithm of minimum angle, *CRT* central retinal thickness, *IDDM* insulin dependent diabetes mellitus, *HbA1c* glycated hemoglobin in %, *crea* serum creatinine, *anti-VEGF* vascular endothelium growth factor inhibitor (ranibizumab only), *DR* diabetic retinopathy, *NPDR* nonproliferative diabetic retinopathy, *PDR* proliferative diabetic retinopathy, *QPDR* quiescent proliferative diabetic retinopathy

### Visual acuity and central retinal thickness

**Fig. 2:**
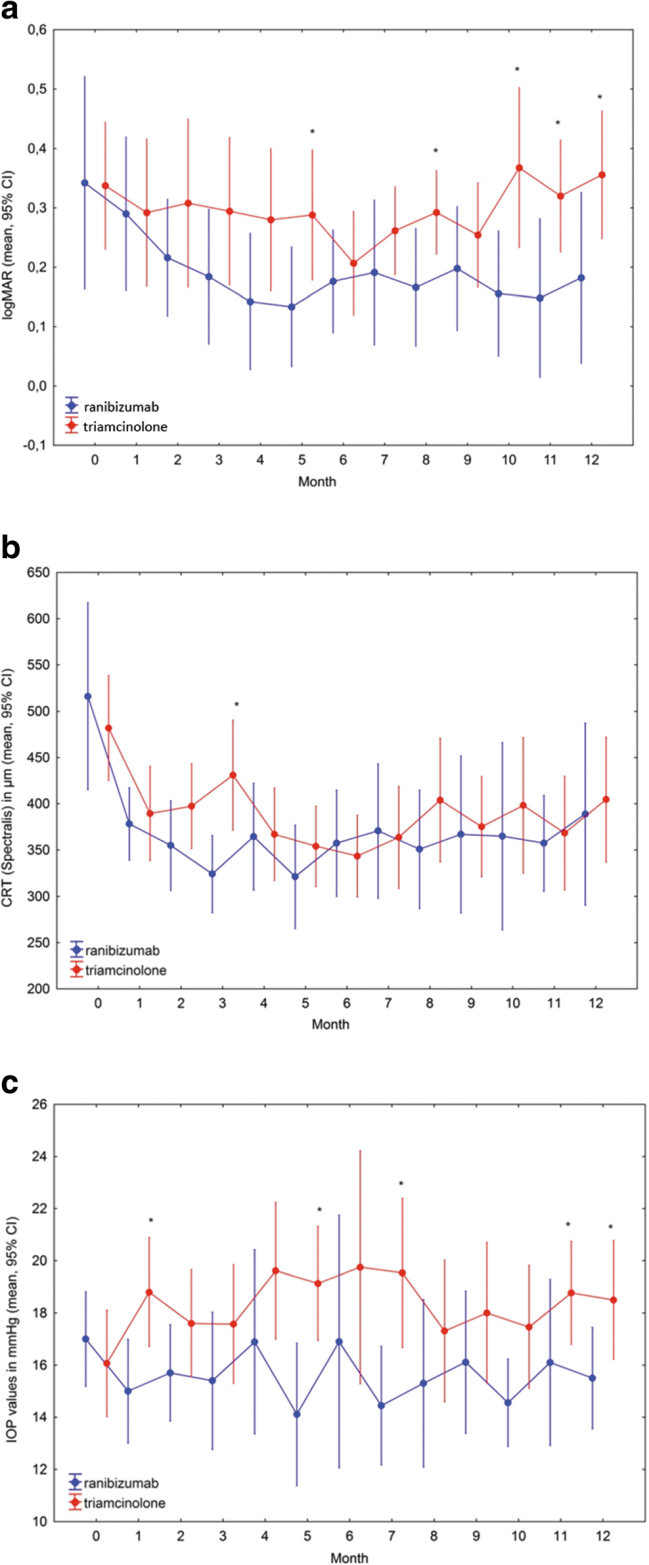
Clinical changes within a year of treatment: Mean best-corrected visual acuity in logMAR (**a**), mean central retinal subfield thickness (CRT, central mm) in μm (**b**) and mean intraocular pressure (IOP) in mmHg (**c**) evaluated monthly over the study period of 12 months. Three months after a single intravitreal injection of 8 mg triamcinolone, edema seems to reoccur, reflected in statistically significant higher CRT at month 3 compared with in the ranibizumab group. Although CRT seems to be similar in the treatment arms at all other time points, patients treated with triamcinolone start to lose vision after 6 months of PRN treatment. Patients treated with triamcinolone had overall higher mean IOP, although baseline values were similar

At baseline, BCVA (ranibizumab logMAR: 0.34 ± 0.25; triamcinolone logMAR: 0.34 ± 0.19; *p* = 0.375) and CRT (ranibizumab: 516 ± 141 μm; triamcinolone: 482 ± 102 μm; *p* = 0.267) were similar in the treatment arms. One month after the first injection, a clinically significant reduction in CRT together with a gain in BCVA was found in both treatment arms [mean change: −138 ± 113 μm (p = <0.001) and logMAR -0.05 ± 0.1 (*p* = 0.07) in the ranibizumab group and −92.4 ± 95.1 μm (p = <0.001) and logMAR -0.05 ± 0.15 (*p* = 0.211) in the triamcinolone group]. This initial effect could be maintained within the first 3 months in both treatment arms. However, while CRT tended to decrease with each ranibizumab injection of the loading dose, CRT gradually, but not statistically significantly, increased in the triamcinolone group receiving sham injections at months 1 and 2 (Fig. 2). At month 3, BCVA was similar in the treatment arms (ranibizumab logMAR: 0.18 ± 0.25; triamcinolone logMAR: 0.21 ± 0.25; *p* = 0.093), while CRT was significantly thicker in the triamcinolone group (ranibizumab: 324 ± 58 μm; triamcinolone: 431 ± 103 μm; *p* = 0.019). During the 1-year follow-up, eyes treated with ranibizumab had significantly thinner CRT at all time points compared with baseline. In eyes treated with triamcinolone, DME recurrence was observed at month 3 (*p* = 0.089 compared with baseline) and month 12 (*p* = 0.055 compared with baseline). While in the ranibizumab group BCVA was significantly better than at baseline at all time-points after the second injection, patients treated with triamcinolone had significantly better visual function at months 4, 6 and 7 only compared with baseline. After 6 months, BCVA stabilized in patients receiving ranibizumab, but progressively decreased in those receiving triamcinolone.

### Characteristics in OCT

**Fig. 3:**
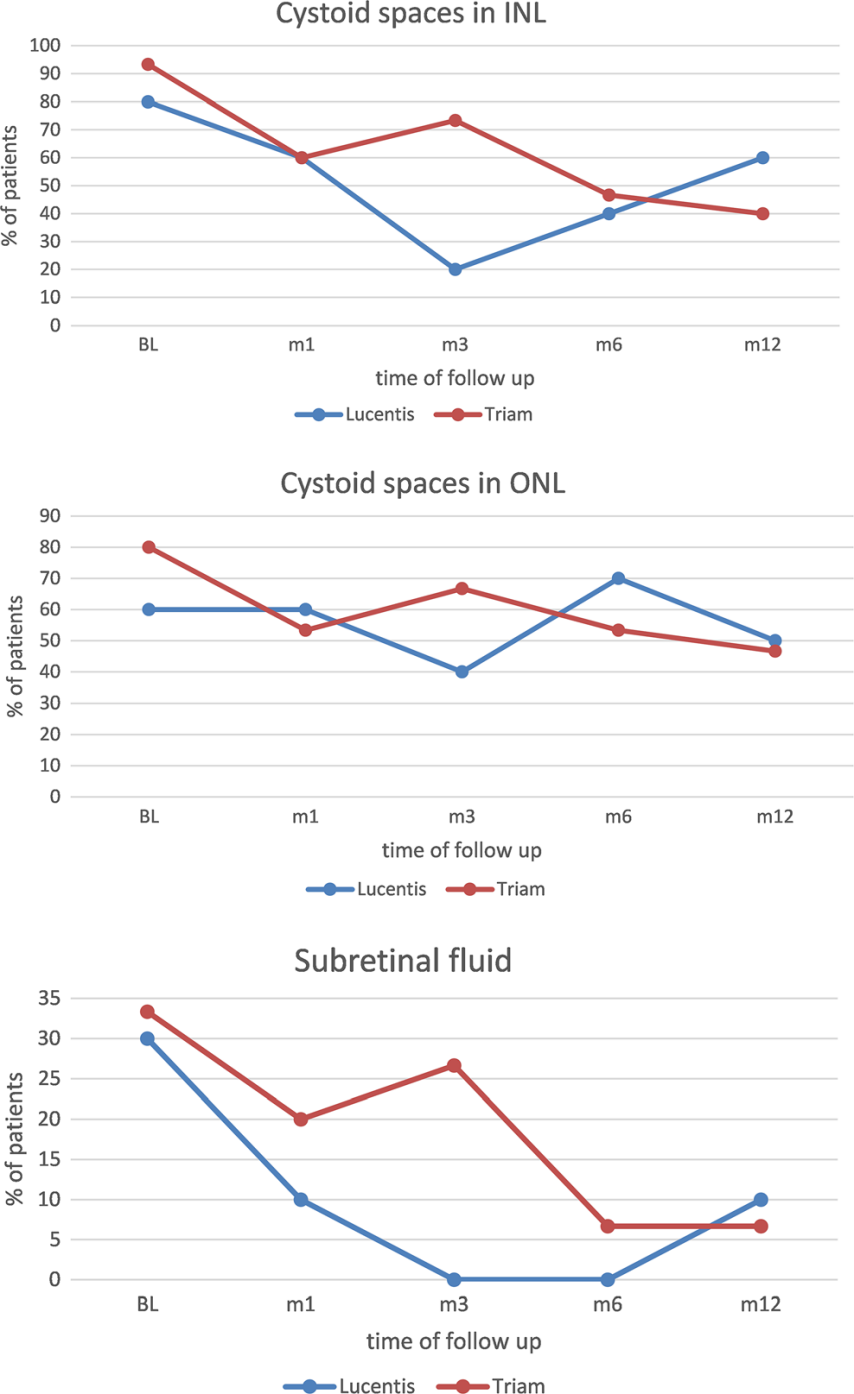
Presence of morphologic changes graded in OCT images: All characteristics tend to reappear 3 months after a single intravitreal triamcinolone injection. Cystoid spaces in the outer nuclear layer (ONL) seem be more persistent than those seen in the inner nuclear layer (INL). Subretinal fluid resolves after repeated injections

At baseline, cystoid spaces in INL or ONL were present in all patients, while four patients (40%) in the ranibizumab arm and 11 (73%) in the triamcinolone arm presented with cystoid spaces in both layers (Table 2). Subretinal fluid was present in about a third of patients in both treatment arms. A positive effect on resolution of cystoid spaces and subretinal fluid was also reflected in the thinning of central retinal thickness that could be observed after the initial injection in both treatment arms. After 3 months the positive effect could be extended by repeated ranibizumab injections, while the effect of a single triamcinolone injection at baseline did not prevail (Fig. 3). Overall, cystoid spaces in the INL responded better than thickening in the ONL to monthly ranibizumab treatment. A recurrence of cystoid spaces in the INL was observed after the loading dose once PRN treatment with ranibizumab started.

**Table 2:** OCT and color fundus photography (CF) characteristics at baseline

OCT + CF characteristics n (%)	Triamcinolone (n = 15)	Ranibizumab (n = 10)
INL cystoid changes	14 (93)	8 (80)
ONL cystoid changes	12 (80)	6 (60)
INL + ONL cystoid changes	11 (73)	4 (40)
Serous retinal detachment	5 (33)	3 (30)
Hard exudates	8 (53)	4 (40)
Cotton wool spots	1 (7)	6 (60)

Cystoid changes in the INL were graded most frequently in both treatment arms. Cotton wool spots were present in six out of ten patients of the ranibizumab group but only in one patient of the triamcinolone group. Hard exudates were present in about half of the patients in both groups

*OCT* optical coherence tomography, *INL* inner nuclear layer, *ONL* outer nuclear layer

### Characteristics in FA

The leakage patterns graded in FA images are summed up in Table 3. Signs of all three leakage patterns were present in three patients (30%) in the ranibizumab group and in two patients (13%) in the triamcinolone group. The source of leakage in most patients was mainly microaneurysms.

**Table 3:** Fluorescence angiography (FA) characteristics

FA characteristics	Triamcinolone (n = 15)	Ranibizumab (n = 10)
Size of FAZ mm2 (±SD)		
Baseline	0.36 (0.15)	0.32 (0.19)
M 12	0.41 (0.15)	0.34 (0.18)
Area of leakage mm^2^ (±SD)		
Baseline	15.13 (11.92)	32.5 (22.34)
M 12	9.63 (8.72)	27.14 (24.17)
Source of leakage (%)		
Mainly from MA (>50%)	87	90
Hardly from MA (<50%)	13	10
Cystic Pattern (%)		
Petaloid	53	70
Honeycomb	47	30
Diffuse	87	90

Early phase FA images were graded for the size of the foveal avascular zone (**FAZ**) by outlining the inner capillaries manually. Late phase FA images were graded for the area of leakage that was outlined manually. The area of leakage was significantly different between both treatment arms at baseline and at month 12. The source of leakage was assessed in early phase images in conjunction with late phase images using a modified grading based on the ETDRS report nr 11. In late phase FA images, the leakage pattern was evaluated

*FAZ* foveal avascular zone, *SD* standard deviation, *MA* microaneurysm

**Fig. 4:**
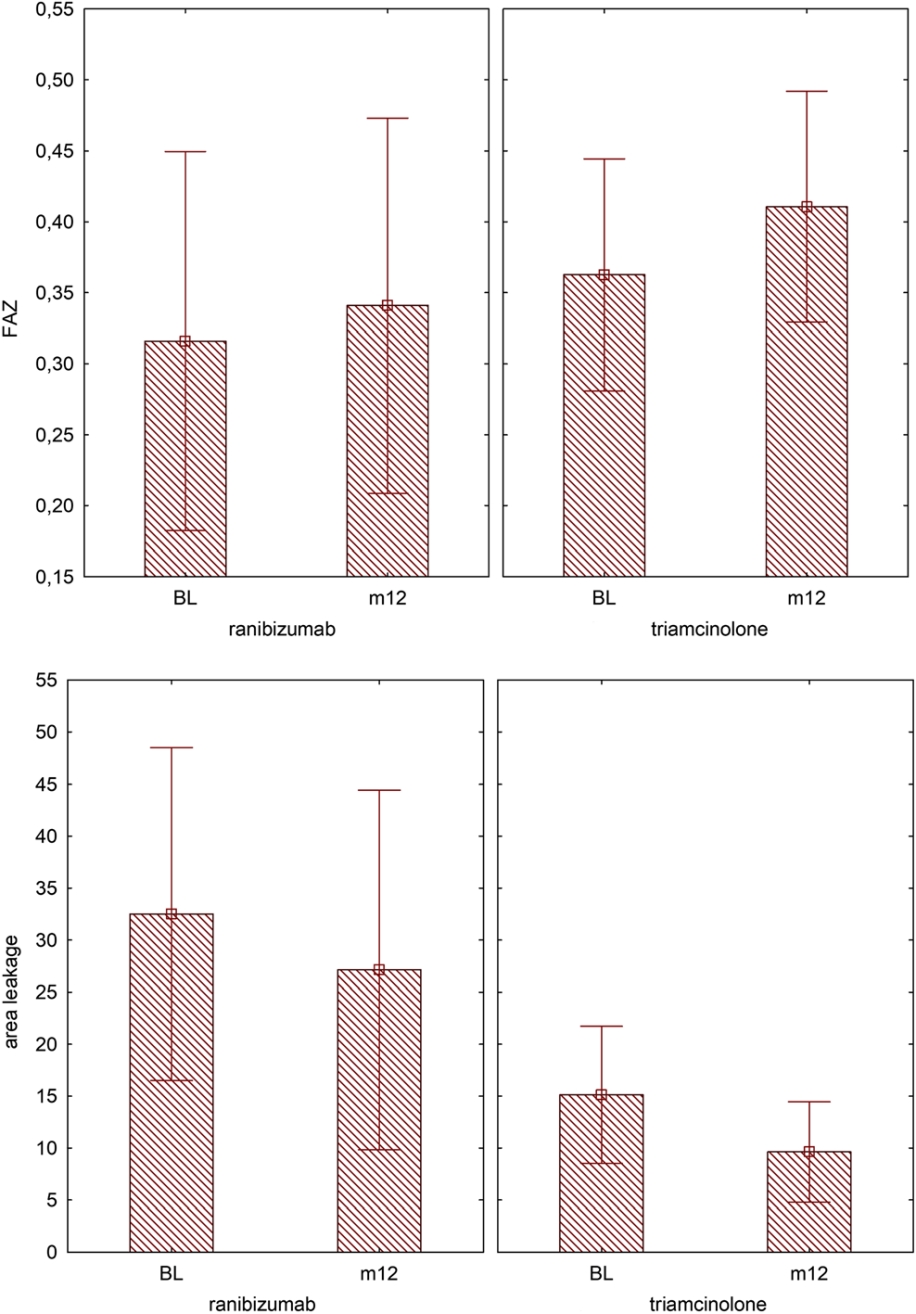
Characteristics graded in fluorescein angiography images: The size of the foveal avascular zone (FAZ) remains stable over the period of treatment while the area of leakage becomes smaller. Although characteristics in OCT were similar in both groups (Fig. 3), the triamcinolone group had a statistically significantly smaller mean area of leakage at baseline and month 12 compared with the ranibizumab group

At baseline the mean size of the foveal avascular zone was 0.32 ± 0.19 mm^2^ in the ranibizumab group and 0.36 ± 0.15 mm^2^ in the triamcinolone group (*p* = 0.493). Within the course of 12 months of PRN treatment, the mean size of the FAZ faintly increased to 0.34 ± 0.18 mm^2^ (*p* = 0.468) in the ranibizumab arm and 0.41 ± 0.15 mm^2^ (*p* = 0.333) in the triamcinolone arm. There was no significant difference between the treatment arms. (Fig. 4, Table 4).

**Table 4:** Analysis of variance (ANOVA) for repeated measures (baseline and month 12), sigma restricted parameterized hypothesis decomposition of the foveal avascular zone (FAZ). There was neither a significant difference between both treatment arms nor between both timepoints

ANOVA FAZ					
	SQ	FG	MQ	F	p
Constant	6,137,560	1	6,137,560	150,2108	0,000000
Therapy	0,040600	1	0,040600	0,9937	0,329,223
Error	0,939,772	23	0,040860		
BL-M12	0,015987	1	0,015987	1,2691	0,271,562
BL-M12*therapy	0,001587	1	0,001587	0,1260	0,725,875
Error	0,289,745	23	0,012598		

The area of leakage was 32.5 ± 22.34 mm^2^ in the ranibizumab and 15.13 ± 11.92 mm^2^ in the triamcinolone group (*p* = 0.012) at baseline. After 12 months of PRN treatment the area of leakage decreased similarly in both treatment arms (*p* = 0.019) (Fig. 4, Table 5).

**Table 5:** **Analysis of variance (ANOVA)** for repeated measures (baseline and month 12) sigma restricted parameterized hypothesis decomposition of the area of leakage in fluorescence angiography. A significant difference was seen at baseline and at month 12 between both treatment arms (bold numbers). The area of leakage regressed significantly within the 12 months independently of the treatment assigned

ANOVA area of leakage					
	SQ	FG	MQ	F	p
Constant	21,368,39	1	21,368,39	42,68,349	0,000001
Therapy	3649,70	1	3649,70	7,29,030	**0,012777**
Error	11,514,36	23	500,62		
BL-M12	354,30	1	354,30	6,32,581	**0,019338**
BL-M12*therapy	0,06	1	0,06	0,00110	0,973,823
Error	1288,19	23	56,01		

### Characteristics in color fundus photography

**Fig. 5:**
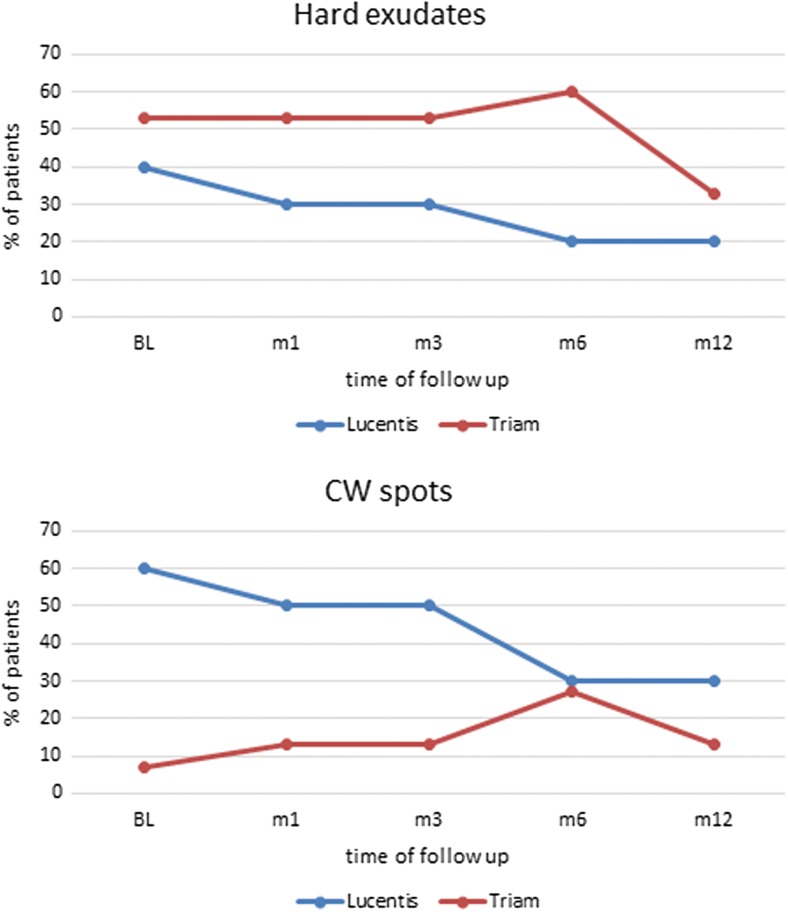
Characteristics graded in color fundus photos: Hard exudates were present in about half of the patients at baseline and slowly decreased over the course of treatment in both treatment arms. Cotton wool spots (CWS) were present in six out of ten patients in the ranibizumab arm at baseline but only in one patient in the triamcinolone arm although signs of ischemia reflected in the size of the foveal avascular zone (Fig. 4) were similar in both groups

The DR severity grading at baseline is summarized in Table 1. At baseline, hard exudates were present in four patients in the ranibizumab group and eight in the triamcinolone group. The presence of hard exudates decreased in both treatment arms over the course of treatment (Fig. 5). CWS by contrast were graded more often in the ranibizumab group at baseline. While they seemed to disappear within 12 months in this group they seemed to be constantly present in the triamcinolone group.

### Number of injections

The mean number of injections needed was 5.9 ± 2.5 out of the 12 possible in the ranibizumab group and 3.2 ± 1.4 out of the four possible in the triamcinolone group. The number of injections necessary was higher during the first 6 months of treatment (mean 4.3 ± 1.1 out of 6 in the ranibizumab group and 1.9 ± 0.4 out of 2 in the triamcinolone group) than in the last 6 months (mean 1.6 ± 1.9 out of 6 in the ranibizumab group *p* = 0.007 and 1.3 ± 1.3 out of 2 in the triamcinolone group *p* = 0.11). Also, 27% of the patients treated with triamcinolone and 50% treated with ranibizumab did not need any further injections after the first 6 months.

### Elevation of intraocular pressure

Clinically significant IOP elevation compared with baseline was observed as early as 1 month after the first intravitreal injection in the triamcinolone group (p = <0.001) and remained above the average of the ranibizumab group throughout the study period of 12 months (Fig. 2). IOP >25 mmHg was measured at least once during follow-up visits in eight patients, of whom seven had been treated with triamcinolone. All of these patients could be managed with topical IOP-lowering medications.

### Cataract progression

Three eyes, two of which received triamcinolone, were pseudophakic. Eight patients (seven treated with triamcinolone and one with ranibizumab) underwent cataract surgery within 12 months after the end of the study.

### Adverse events

Ocular adverse events such as ocular discomfort after intravitreal injection or a rise in IOP in the triamcinolone group were anticipated (Fig. 2). There was no case of endophthalmitis or retinal detachment.

## Discussion

Our data on the overall treatment response assessed as a change in BCVA and CRT are in accordance with previous studies, which have proven the clinical efficacy of ranibizumab and triamcinolone for DME therapy [16, 17]. Consistent with the data from Protocol I of the DRCR, the net initial gain in visual acuity in patients treated with triamcinolone was lost within the second half of the year [18]. Even though we used a double dose of triamcinolone (8 mg instead of 4 mg in Protocol I) and the minimum treatment interval was shorter (12 instead of 16 weeks), the initial effect on DME resolution in the triamcinolone arm did not prevail as long as expected. After 3 months, patients treated with ranibizumab had a significantly thinner CRT than those treated with triamcinolone (*p* = 0.019). Similar peaks of edema recurrence were seen in Protocol I at 16, 32 and 48 weeks after initial triamcinolone injection accordingly. The fading potency of a single intravitreal triamcinolone injection was also reflected in the recurrence of SRF and cystoid spaces after 3 months. Hence, reinjection of triamcinolone should be considered earlier in some patients, depending on morphologic dynamics, to avoid under-treatment.

Overall, CRT was similar in the treatment arms at baseline and at month 12 (*p* = 0.271 and *p* = 0.426, respectively), but BCVA was significantly better in the ranibizumab group after 12 months (*p* = 0.015). A decrease in BCVA after 6 months of triamcinolone treatment is consistent with data published in a previous paper, where the loss in BCVA was attributed to cataract formation because pseudophakic patients treated with triamcinolone and those treated with ranibizumab had similar visual acuity results [18]. As cataracts were not to be operated during the study period, loss of BCVA in the triamcinolone-treated eyes might have been related to cataract formation. In our cohort there were only two pseudophakic patients in the triamcinolone treatment arm, so we could not do a subgroup evaluation. As has been reported in previous studies, intravitreally applied triamcinolone is associated with an increased risk of IOP elevation [19]. Although seven out of 15 patients (47%) in the triamcinolone group had at least one IOP measurement >25 mmHg compared with only one (10%) in the ranibizumab group, all patients responded well to IOP-lowering medication.

We focused on morphological details graded in OCT and FA images because the overall treatment response assessed as changes in BCVA and CRT after anti-VEGF or triamcinolone application in diabetic macular edema has already been studied extensively. OCT characteristics have been shown to be predictive for the treatment response in patients with age-related macular degeneration [20]. In general, morphologic features in DME have been described in detail and some characteristics graded at baseline found to be associated with a better treatment response to DME therapy [21–24]. Nevertheless, a continuous evaluation of anatomic characteristics in OCT and FA images over the course of 1 year of treatment with ranibizumab or triamcinolone has not yet been published. Patients with intraretinal cysts and preservation of the foveal contours have been found to benefit most from intravitreal bevacizumab therapy in terms of BCVA gain [25]. In our cohort, INL cysts disappeared in six out of eight patients (75%) after 3 monthly injections of ranibizumab, while ONL swelling seemed to be more persistent. However, patients treated with ranibizumab gained statistical significance in BCVA within the first 3 months of treatment. These results partially contrast with those of another study where the gain in visual acuity was mainly attributed to the regression of ONL cysts [26]. However, the morphologic evaluation in that study was limited to three trans-foveal OCT scans, whereas we evaluated the presence of ONL swelling within all 49 OCT scans of the macular cube.

In addition to INL and ONL cysts, serous retinal detachment was graded in 30% of patients. Both treatment strategies were effective in serous retinal detachment reduction after repeated intravitreal application. Fluctuations over time paralleled the trend of cystoid intraretinal fluid accumulation. Three out of ten patients in the ranibizumab group and one out of 15 in the triamcinolone group did not need any further treatment after the first 3 months over the 12 months study duration. Morphologic characteristics at baseline were similar to those of the patients who needed repeated treatment within the study period. Hence, we could not identify any morphologic characteristics that predicted the overall treatment response in our study patients.

Interestingly, the area of leakage measured in FA images was statistically significantly different at baseline, although the source of leakage, size of FAZ and OCT characteristics were similar between the two groups. However, the area of leakage statistically significantly regressed in both treatment arms (Fig. 4). There is evidence that fluorescein leakage without intraretinal fluid accumulation in OCT is related to the breakdown of the RPE barrier and that neutralizing VEGF with an antibody leads to a partial recovery of barrier properties [27, 28]. The importance of the RPE pump function in the pathogenesis and treatment response in patients with DME is still unknown. Therefore, we cannot exclude the possibility that the difference in leakage area at baseline was a confounding factor in our study.

The major drawback of our study was the single center design entailing a relatively small number of study patients. Nevertheless, the prospective, randomized, double-masked study design, the multimodal retinal imaging techniques including regularly obtained FA and SD OCT images and the thorough masked image analyses of the whole macula add to the value of data presented.

To conclude, this descriptive analysis of morphologic characteristics in DME emphasizes the high diversity of diabetic macular edema that cannot be reflected in central retinal thickness alone. Furthermore, it gives a detailed insight into the treatment response to two different agents over 1 year. Yet predicting morphological characteristics has not been accomplished. In the future, detailed retinal image analyses might add to individualized treatment strategies.

## References

[1] JWY Y, Rogers SL, Kawasaki R (2012). Global prevalence and major risk factors of diabetic retinopathy. Diab Care.

[2] Collaboration N risk factor (2008). Worldwide trends in diabetes since 1980 : a pooled analysis of 751 population-based studies with 4 · 4 million participants. Lancet.

[3] Murakami T, Yoshimura N (2013). Structural changes in individual retinal layers in diabetic macular edema. J Diab Res.

[4] Early Treatment Diabetic Retinopathy Study Research Group (1991). Classification of diabetic retinopathy from fluorescein angiograms. ETDRS report number 11. Ophthalmology.

[5] Otani T, Kishi S, Maruyama Y (1999). Patterns of diabetic macular edema with optical coherence tomography. Am J Ophthalmol.

[6] Bolz M, Lammer J, Deak G (2014). SAVE: a grading protocol for clinically significant diabetic macular oedema based on optical coherence tomography and fluorescein angiography. Br J Ophthalmol.

[7] Soliman W, Sander B, Hasler PW, Larsen M (2008). Correlation between intraretinal changes in diabetic macular oedema seen in fluorescein angiography and optical coherence tomography. Acta Ophthalmol.

[8] Elman MJ, Ayala A, Bressler NM (2015). Intravitreal ranibizumab for diabetic macular edema with prompt versus deferred laser treatment: 5-year randomized trial results. Ophthalmology.

[9] Schmidt-Erfurth U, Lang GE, Holz FG (2014). Three-year outcomes of individualized ranibizumab treatment in patients with diabetic macular edema: the RESTORE extension study. Ophthalmology.

[10] The Diabetic Retinopathy Clinical Research Network (2015). Aflibercept, Bevacizumab, or Ranibizumab for Diabetic Macular Edema. N Engl J Med.

[11] Kriechbaum K, Prager S, Mylonas G (2014). Intravitreal bevacizumab (Avastin) versus triamcinolone (Volon A) for treatment of diabetic macular edema: one-year results. Eye (London, England).

[12] Bandello F, Tejerina AN, Vujosevic S (2015). Retinal layer location of increased retinal thickness in eyes with subclinical and clinical macular edema in diabetes type 2. Ophthalmic Res.

[13] Heng LZ, Pefianaki M, Hykin P, Patel PJ (2015) Interobserver agreement in detecting spectral- domain optical coherence tomography features of diabetic macular edema. 1–8 10.1371/journal.pone.0126557 PMC4440774 25996150

[14] Deák GG, Bolz M, Ritter M (2010). A systematic correlation between morphology and functional alterations in diabetic macular edema. Invest Ophthalmol Vis Sci.

[15] Otani T, Kishi S (2007). Correlation between optical coherence tomography and fluorescein angiography findings in diabetic macular edema. Ophthalmology.

[16] Elman MJ, Ayala A, Bressler NM, et al (2014) Intravitreal Ranibizumab for diabetic macular edema with prompt versus deferred laser treatment: 5-year randomized trial results. Ophthalmology 1–7 10.1016/j.ophtha.2014.08.047 PMC4520307 25439614

[17] Gillies MC, McAllister IL, Zhu M (2011). Intravitreal triamcinolone prior to laser treatment of diabetic macular edema: 24-month results of a randomized controlled trial. Ophthalmology.

[18] Elman M, Aiello L, Beck R, Bressler N (2010). Randomized trial evaluating ranibizumab plus prompt or deferred laser or triamcinolone plus prompt laser for diabetic macular edema. Ophthalmology.

[19] Gillies MC, Lim LL, Campain A (2014). A Randomized Clinical Trial of Intravitreal Bevacizumab versus Intravitreal Dexamethasone for Diabetic Macular Edema The BEVORDEX Study.

[20] Waldstein SM, Simader C, Staurenghi G (2016). Morphology and visual acuity in Aflibercept and Ranibizumab therapy for Neovascular age-related macular degeneration in the VIEW trials. Ophthalmology.

[21] Horii T, Murakami T, Nishijima K (2010). Optical coherence tomographic characteristics of microaneurysms in diabetic retinopathy. Am J Ophthalmol.

[22] Bolz M, Schmidt-Erfurth U, Deak G (2009). Optical coherence tomographic hyperreflective foci: a morphologic sign of lipid extravasation in diabetic macular edema. Ophthalmology.

[23] Soliman W, Sander B, Jørgensen TM (2007). Enhanced optical coherence patterns of diabetic macular oedema and their correlation with the pathophysiology. Acta Ophthalmol Scand.

[24] Sophie R, Lu N, Campochiaro PA (2015). Predictors of functional and anatomic outcomes in patients with diabetic macular edema treated with Ranibizumab. Ophthalmology.

[25] Cheema HR, Al Habash A, Al-Askar E (2014). Improvement of visual acuity based on optical coherence tomography patterns following intravitreal bevacizumab treatment in patients with diabetic macular edema. Int J Ophthalmol.

[26] Reznicek L, Cserhati S, Seidensticker F (2013). Functional and morphological changes in diabetic macular edema over the course of anti-vascular endothelial growth factor treatment. Acta Ophthalmol.

[27] Weinberger D, Fink-Cohen S, Gaton D (1995). Non- retinovascular leakage in diabetic maculopathy. D, Weinberger S, fink-Cohen DD, Gaton E, Priel Y., Yassur. Br J Ophthalmol.

[28] Hartnett M, Lappas A, Darland D et al (2003) Retinal pigment epithelium and endothelial cell inter- action causes retinal pigment epithelial barrier dysfunction via a soluble VEGF-dependent mechanism. Exp Eye Res:593–599 10.1016/s0014-4835(03)00189-1 14550401

